# Tractography of the parahippocampal gyrus and material specific memory impairment in unilateral temporal lobe epilepsy

**DOI:** 10.1016/j.neuroimage.2007.12.046

**Published:** 2008-05-01

**Authors:** M. Yogarajah, H.W.R. Powell, G.J.M Parker, D.C. Alexander, P.J. Thompson, M.R. Symms, P. Boulby, C.A Wheeler-Kingshott, G.J. Barker, M.J. Koepp, J.S. Duncan

**Affiliations:** a Department of Clinical and Experimental Epilepsy, Institute of Neurology, UCL and National Society for Epilepsy, UK; b Imaging Science and Biomedical Engineering, University of Manchester, UK; c Department of Computer Science, UCL, UK; d Department of Neuroinflammation, Institute of Neurology, UCL, UK; e Department of Clinical Neuroscience, Centre for Neuroimaging Sciences, King''s College London, Institute of Psychiatry, UK

**Keywords:** Tractography, Parahippocampal gyrus, Memory, Epilepsy

## Abstract

**Introduction:**

Temporal lobe epilepsy (TLE) is associated with disrupted memory function. The structural changes underlying this memory impairment have not been demonstrated previously with tractography.

**Methods:**

We performed a tractography analysis of diffusion magnetic resonance imaging scans in 18 patients with unilateral TLE undergoing presurgical evaluation, and in 10 healthy controls. A seed region in the anterior parahippocampal gyrus was selected from which to trace the white matter connections of the medial temporal lobe. A correlation analysis was carried out between volume and mean fractional anisotropy (FA) of the connections, and pre-operative material specific memory performance.

**Results:**

There was no significant difference between the left and right sided connections in controls. In the left TLE patients, the connected regions ipsilateral to the epileptogenic region were found to be significantly reduced in volume and mean FA compared with the contralateral region, and left-sided connections in control subjects. Significant correlations were found in left TLE patients between left and right FA, and verbal and non-verbal memory respectively.

**Conclusion:**

Tractography demonstrated the alteration of white matter pathways that may underlie impaired memory function in TLE. A detailed knowledge of the integrity of these connections may be useful in predicting memory decline in chronic temporal lobe epilepsy.

## Introduction

Temporal lobe epilepsy (TLE) is the most common form of refractory focal epilepsy (Crawford, 2000). Parahippocampal structures, which are critically implicated in the generation and propagation of seizures in TLE (Avoli et al., 2002; Bertram, 2006; Du et al., 1993; Plate et al., 1993; Rutecki et al., 1989; Spencer and Spencer, 1994), are essential for declarative memory (Eichenbaum, 2000). Longitudinal neuropsychological studies have shown that persisting epilepsy is associated with progressive memory impairment (Dodrill, 2002; Helmstaedter et al., 2003). Those who undergo anterior temporal lobe resection (ATLR) are at further risk of memory impairment, the nature of which depends on whether surgery is on the dominant or non-dominant side (Ivnik et al., 1987; Spiers et al., 2001).

Neuropsychological assessment, quantitative MRI, and latterly functional MRI (fMRI) indicate the role of the medial temporal lobe structures (MTL) in sustaining material specific memory functions (Powell et al., 2005), and the reorganisation of memory that occurs with TLE (Powell et al., 2007a). These and lesion deficit studies (Frisk and Milner, 1990; Smith and Milner, 1981) have shown that memory deficit after ATLR is related to the *functional* integrity of the parahippocampal structures. The strength of connections, or structural connectivity, of the parahippocampal gyrus in TLE has not been evaluated, or related to function.

Tractography is a technique which uses diffusion tensor imaging (DTI) data to delineate white matter tracts, and to quantify their volume, and infer structural characteristics (Johansen-Berg and Behrens, 2006). Using one such tractography technique, Probabilistic Index of Connectivity or PICo (Parker et al., 2003; Parker and Alexander 2003), we evaluated the structural connectivity of the parahippocampal gyrus in TLE, with the hypothesis that this would be impaired ipsilaterally to the seizure focus, and that the degree of any such impairment would correlate with material specific memory function.

## Methods

### Subjects

We studied 18 patients (median age 33.5 years; range 22–47 years; 11 males) with medically refractory TLE undergoing pre-surgical evaluation at the National Hospital for Neurology and Neurosurgery, London, UK. All patients had undergone structural MRI at 1.5T (Duncan, 1997). Of the eight left TLE patients, seven had hippocampal sclerosis (HS) (one also had a ganglioglioma in the left fusiform gyrus) and one had a MTL dysembryoblastic neuroepithelial tumour (DNET). Of the ten right TLE patients, seven had HS, one had a right MTL glioma, another had a right MTL DNET, and the other had right superior temporal focal cortical dysplasia (FCD). Video-EEG had confirmed seizure onset in the MTL ipsilateral to the clinically defined seizure site, and all patients had a normal, contralateral hippocampus based on qualitative and quantitative MRI criteria (Woermann et al., 1998).

All patients were on anti-epileptic medication, and were fluent English language speakers. Handedness was determined using the Edinburgh handedness inventory (Oldfield, 1971), and language dominance was determined using a range of fMRI tasks which have been described previously and include the use of verbal fluency, and reading tasks (Powell et al., 2006). All patients underwent a standardised pre-surgical neuropsychological assessment (Baxendale et al., 1998). Patient demographics, neurological test results, and surgical outcome data are listed in Table 1. The ILAE classification of post-operative seizure outcome following epilepsy surgery was used (Wieser et al., 2001). We also studied 10 right-handed native English speaking, healthy volunteers (median age 29.5 years; range 23 to 50 years; 7 females). The study was approved by the National Hospital for Neurology and Neurosurgery and the Institute of Neurology Joint Ethics Committee, and informed written consent was obtained from all subjects.

### MR data acquisition

MRI studies were performed on a 1.5-T GE Signa Horizon scanner (General Electric, Wakashua, Milwaukee, Wisconsin, USA). Standard imaging gradients with a maximum strength of 22 m Tm^- 1^ and slew rate 120 Tm^- 1^s^- 1^ were used. All data were acquired using a standard quadrature birdcage head coil for both RF transmission and reception. The scanning protocol also included a coronal T1-weighted volumetric acquisition sequence with 1.5-mm-thick slices, and hippocampal volumes were determined according to a previously described method (Moran et al., 1999).

### Diffusion tensor imaging

The DTI acquisition sequence was a single-shot spin-echo planar imaging (EPI) sequence, cardiac gated (triggering occurring every QRS complex) (Wheeler-Kingshott et al., 2002), with TE = 95 ms. Sets of 60 contiguous 2.3-mm thickness axial slices were obtained, covering the whole brain, with diffusion sensitizing gradients applied in each of 54 non-colinear directions (maximum *b* value of 1148 mm^2^ s^- 1^ (*δ* = 34 ms, Δ = 40 ms, using full gradient strength of 22 mTm^- 1^)) along with 6 non-diffusion weighted (*b* = 0) scans. The field of view was 24 cm, and the acquisition matrix size was 96 × 96, zero filled to 128 × 128 during reconstruction so that the reconstructed voxel size was 1.8 × 1.8 × 2.3 mm^3^. The DTI acquisition time for a total of 3600 image slices was approximately 25 min (depending on the heart rate).

We used the method of Parker and Alexander (Parker et al., 2003; Parker and Alexander, 2003) to reduce fibre orientation ambiguities in voxels containing fibre crossings. Voxels in which the single tensor fitted the data poorly were identified using the spherical-harmonic voxel-classification algorithm of Alexander et al. (Alexander et al., 2002). In these voxels a mixture of two Gaussian probability densities was fitted and the principal diffusion directions of the two diffusion tensors provided estimates of the orientations of the crossing fibres (Tuch et al., 2002). In all other voxels a single tensor model was fitted. For all voxels, fractional anisotropy (FA) maps were generated from the single tensor fit (Pierpaoli et al., 1996; Pierpaoli and Basser, 1996).

### Tractography

All scans were transferred to a Unix workstation for processing. We used the PICo algorithm extended to cope with crossing fibres (Parker et al., 2003; Parker and Alexander, 2003) to track from anatomically defined regions of interest (ROIs) within the parahippocampal gyrus. This algorithm adapts the commonly used streamline approach to exploit the uncertainty due to noise in one or more fibre orientations defined for each voxel. This uncertainty is defined using probability density functions (PDFs) constructed using simulations of the effect of realistic data noise on fibre directions obtained from the mixture model (Parker and Alexander, 2003). The streamline process is repeated using Monte Carlo methods to generate maps of connection probability or confidence of connection from the chosen start region(s).

**Fig. 1:**
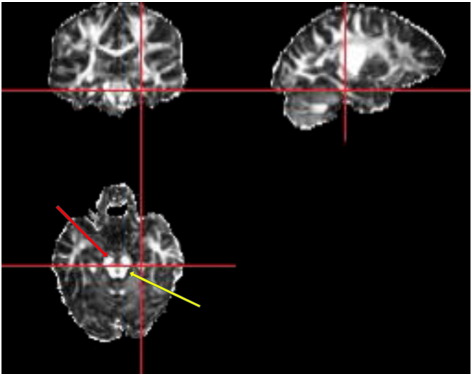
Axial, coronal and sagittal views of a single subject FA map. The red arrow points towards the cerebral peduncles, and the yellow arrow points towards the brainstem. The white matter of the parahippocampal gyrus is readily identifiable in the crosshairs in all three corresponding views.

The anatomical definition of the ROI was based on a previously published tractography analysis of the parahippocampal gyrus in healthy subjects (Powell et al., 2004). Viewing the FA images in three orthogonal planes using MRIcro (http://www.psychology. nottingham.ac.uk), the centre of the white matter tract just anterior to the brainstem, and posterior to the cerebral peduncles was selected, such that the parahippocampal gyrus was defined at its longest in the corresponding sagittal view (Fig. 1). The corresponding coronal slice was then used to select two adjacent voxels in a left-right direction, such that they both lay within the white matter tract on axial and sagittal views. This process was repeated in one anterior and one posterior coronal slice. This method was chosen as the parahippocampal gyrus runs anterior to posterior, inferior to superior and medial to lateral within the medial temporal lobe. The principal eigenvector of each voxel, when viewed using PICo and projected on the axial plane was orientated anterior-posterior. A threshold of FA ≥ 0.1, and curvature threshold of 180° were set for tractography.

### Neuropsychological tests

The list learning and design learning tests were used to assess material specific memory function (Coughlan and Hollows, 1985). In the verbal learning task the subject is read a list of 15 words five times, and on each presentation attempts to recall as many of the words as possible. The overall percentage of correct responses was used as the measure of verbal memory efficiency. For non-verbal memory we employed a design learning task; the subject is presented with a visual design on five occasions with recall being tested after each presentation. The percentage of correct responses over the five trials was used as a second measure of non-verbal memory efficiency. These tests form part of our presurgical memory assessment in TLE cases, and have proven least affected by performance anxiety, have a good test-retest reliability and are sensitive indicators of medial temporal lobe function (Baxendale et al., 1998). Neuropsychological test results are listed in Table 2.

### Data analysis

All data were analysed using SPSS (11.0.0). It was first verified whether all parameters were normally distributed using the Kolmogorov–Smirnov test for normal distribution. Group differences for age were determined by a one-way analysis of variance (ANOVA), and gender distribution was assessed using the Pearson's *χ*^2^ test. The age of onset of epilepsy, duration of epilepsy, and frequency of complex partial (CPS) and secondarily generalized seizures (SGS) in right and left TLE patients were compared using the Mann–Whitney *U* test.

Each subject's output tractography connection probability map was spatially normalised by mapping into a standard space using the MNI template provided by SPM2 (Wellcome Department of Imaging Neuroscience, London; http://www.fil.ion.ucl.ac.uk/spm). Binary masks at a threshold connection probability value of 0.05 were then constructed. Our group has previously demonstrated in this same group of patients and control subjects, that the threshold 0.05 strikes a balance between losing non-specific low probability connections, while retaining the main body of the pathways (Powell et al., 2006). Binary masks at this threshold were therefore averaged across each group, to produce variability (or commonality) maps indicating the degree of spatial variability and overlap of the identified connections (Parker et al., 2005). A voxel commonality value C of 1.0 indicates that every individual had a connection identified in this voxel, while a C value of 0 indicates that none of them did (Parker et al., 2005). Tracts were then assessed visually in all three planes for visual symmetry and size/extent using MRIcro.

Normalised tract volumes were calculated for the connecting tracts from the left and right PHG of each control and patient at a threshold of 0.05 (Toosy et al., 2004). An asymmetry index for volume (AI_vol_) defined as AI_vol_ = [100 × (Right Volume − Left Volume)] / [(Right Volume + Left Volume)/2] was calculated (Jutila et al., 2001), and the mean values between groups compared using a one way ANOVA analysis. Comparisons between the control AI values, and left and right TLE AI values were carried out using post-hoc Dunnett *t*-tests. Two way mixed ANOVA with one between subjects factor (group –— controls or TLE (both left and right)) and one within subjects factor (hemisphere — left or right) was used to test for the effect of interaction between group and hemisphere on volume, and unpaired *t*-tests were used to compare the patient and control group tract volumes.

The mean FA of the connected volume was calculated in native space for the left and right tracts in controls and patients. This was carried out by multiplying the native, thresholded, binarised images with that subject's whole brain FA image, in order to calculate the mean intensity value of the voxels. An asymmetry index for FA (AI_FA_) defined as AI_FA_ = [100 × (Right FA - Left FA)] / [(Right FA + Left FA)/2] (Jutila et al., 2001) was calculated, and the mean values between groups compared using one way ANOVA analysis. Comparisons between the control AI values and left and right TLE AI values were carried out using post-hoc Dunnett *t*-tests. Two way mixed ANOVA with one between subjects factor (group — controls or TLE (both left and right)) and one within subjects factor (hemisphere — left or right) were used to test for the effect of an interaction between group and hemisphere on FA, and unpaired *t*-tests were used to compare the patient and control group tract FA values.

Pearson's correlation test was used to evaluate the evidence for a correlation of hippocampal volume with tract volume and FA, and tract volume with tract FA, ipsilateral and contralateral to the seizure focus. Performance on material specific memory measures was investigated for evidence of a correlation with tract volume and FA in right and left TLE groups, omitting patient 14 because of his atypical language dominance.

## Results

### Demographic analysis

There was no significant difference in the mean age or gender distribution of participants in the three groups (controls, left TLE, right TLE). There was no significant difference in the age of onset, duration of epilepsy or frequency of CPS and SGS between left and right TLE patients.

### Qualitative tract analysis

**Fig. 2:**
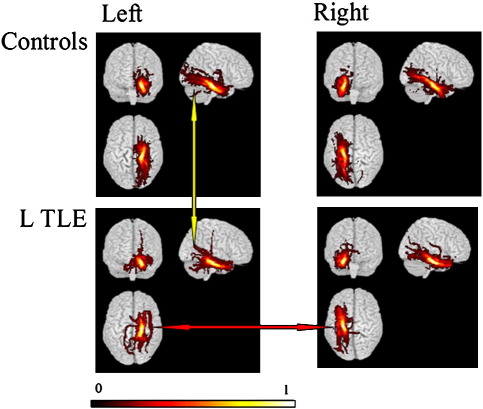
Group maps of the parahippocampal gyrus tracts comparing left TLE patients against controls. Tracts are projected onto a 3d rendered brain template. A voxel commonality value of 1.0 indicates that each individual had a connection identified in this voxel while a value of 0.0 indicates that none of them did. In controls, PHG connections were symmetric. There is more marked asymmetry of connections in left TLE patients compared with controls. There is a decrease in ipsilateral compared with contralateral connections in left TLE, such that the body of the PHG appears smaller (red arrow). In addition, fewer connections to the occipital lobe are present in left TLE patients compared with controls (yellow arrow).

**Fig. 3:**
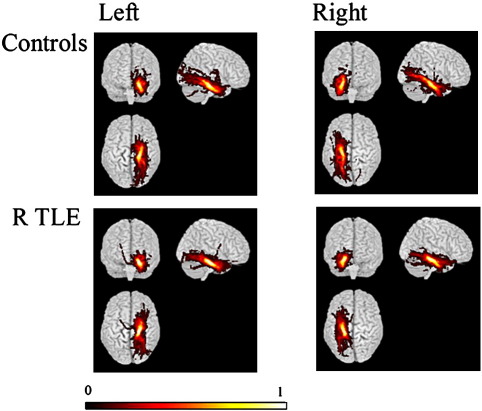
Group maps of the parahippocampal gyrus tracts comparing right TLE patients against controls. Tracts are projected onto a 3d rendered brain template. A voxel commonality value of 1.0 indicates that each individual had a connection identified in this voxel while a value of 0.0 indicates that none of them did. In controls, PHG connections were symmetric. There is more marked asymmetry of connections in left TLE patients compared with controls and right TLE patients compared with Fig. 2.

In controls, PHG connections were visually symmetric. Connections between the para-hippocampal gyrus and anterior temporal lobe, orbitofrontal areas and posterior temporal and extrastriate occipital areas were observed as documented previously (Powell et al., 2004). There was a clear decrease in ipsilateral compared with contralateral connections in left TLE (Fig. 2), though an ipsilateral reduction was not evident in the commonality map of the right TLE group (Fig. 3).

### Quantitative tract analysis

There was a significant difference in AI_vol_ between groups [*F*(2,25) = 3.31 *p* = 0.05]. The AI_vol_ was greater in left TLE patients than in controls (*p* = 0.05) with a mean 22% reduction in volume on the left (Table 3). There was no significant difference between the AI_vol_ in right TLE patients and controls. There was no significant interaction between group and hemisphere for tract volume, and no significant differences between left and right volumes in left/right TLE patients, and controls.

There was a significant difference in AI_FA_ between groups [*F*(2, 25) = 4.92 *p* = 0.02], attributable to reduced FA on the left in left TLE (*p* = 0.02 against controls). There was no significant difference between the AI_FA_ in right TLE patients and controls. There was a significant interaction between group and hemisphere on tract FA [*F*(2, 25) = 4.35 *p* = 0.02], with FA being lower on the left in left TLE patients, compared with controls (*p* = 0.03). Although a similar trend was present in right TLE patients, with FA being lower on the right side compared with controls, this was not significant (*p* = 0.06).

### Correlation of tract volume and FA with pre-surgical material specific memory

**Fig. 4:**
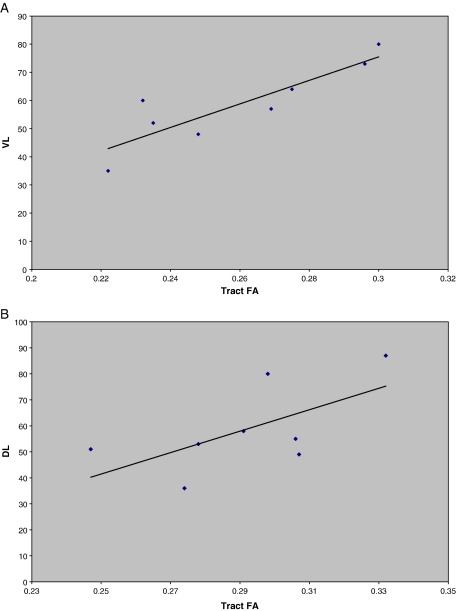
The correlation of material specific memory against left PHG FA in left TLE patients where VL = verbal learning (*r* = 0.876, *p* ≤ 0.05 ) and DL = design learning (*r* = 0.630, *p* ≤ 0.05).

All but one patient were left hemisphere dominant for language. Patient 14 was left handed and right hemisphere dominant on both fMRI and intracarotid amytal testing. He was therefore omitted from the correlation analysis. In the left TLE patients, FA of the left PHG connections were correlated significantly with pre-surgical verbal learning (*r* = 0.88, *p* = 0.002), and right FA correlated significantly against pre-surgical design learning (*r* = 0.63, *p* = 0.05) (Fig. 4). Left and right tract volumes were not significantly correlated with verbal learning and design learning, respectively. In the right TLE patients, there were no significant correlations between left or right tract FA or volume, with pre-operative verbal learning or design learning respectively.

### Correlations of hippocampal volumes with tract volumes and tract FA

There was no significant correlation between ipsilateral hippocampal volume and ipsilateral tract volumes or FA nor between contralateral hippocampal volumes and contralateral tract volume or FA.

### Correlation analysis of tract volume with tract FA

**Fig. 5:**
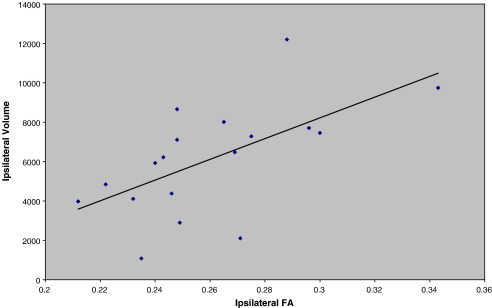
The correlation between FA and volume of the parahippocampal gyrus in TLE patients, ipsilateral to seizure focus (*r* = 0.606, *p* ≤ 0.05).

There was a significant correlation between tract FA and tract volume (*r* = 0.61, *p* = 0.008) ipsilateral to seizure focus (Fig. 5), but not contralateral to seizure focus.

## Discussion

Our principal finding was that in TLE the white matter connections of the parahippocampal gyrus ipsilateral to the seizure focus had smaller volumes and decreased FA. This was statistically significant in left but not right TLE patients. Furthermore, in left TLE, decreased FA was associated with poorer performance on both material specific memory measures. These results are consistent with the hypothesis that TLE involves dysfunction and structural changes in a network that includes the parahippocampal gyrus.

The connections of the human parahippocampal gyrus, visualized with tractography, have been described in a control population (Powell et al., 2004). Using a different tractography algorithm (Fast Marching Tractography or FMT) connectivity was found between the parahippocampal gyrus and the anterior temporal lobe, orbitofrontal areas, posterior temporal lobe and extra-striate occipital lobe via the lingual and fusiform gyri. These findings are similar to those in this control group.

This is the first study to use tractography to quantitatively assess the structural changes in the parahippocampal gyrus in TLE. Several volumetric MRI (Bernasconi et al., 2003; Bonilha et al., 2003) and voxel-based morphometry (VBM) studies (Bernasconi et al., 2004; Bonilha et al., 2004; Keller et al., 2002b,a, 2004) have investigated the extra-hippocampal structural changes in unilateral TLE. The para-hippocampal gyrus or its sub-regions including the entorhinal cortex, parahippocampal cortex and perirhinal cortex have been shown to be affected in TLE. Several studies suggested that the degree of atrophy and regional distribution was more extensive in left TLE, though none were specifically designed to evaluate this (Bernasconi et al., 2004; Bonilha et al., 2004; Keller et al., 2002b). It has however been recently demonstrated that the distribution and severity of extra-hippocampal grey matter loss is more extensive in left TLE patients than right TLE patients (Bonilha et al., 2007). Our study is the first to show that the white matter connections of the parahippocampal gyrus are affected by unilateral TLE. This process appears to be more severe in left TLE compared with right TLE. A similar pattern was observed in another tractography study that assessed language pathways in TLE patients (Powell et al., 2007b), although in this case there was also asymmetry of connections in controls, with more extensive connections in the speech dominant hemisphere. Ultimately, longitudinal quantitative MRI studies will be necessary to determine the effects of underlying cause, seizures, medications, and co-morbidity on the white matter structures and connections of the brain.

### Parahippocampal connections and memory in temporal lobe epilepsy

The memory deficits associated with TLE, particularly left TLE, can be disabling. Consequently, much attention has been focused on the effects of both chronic TLE and ATLR on memory. While the hippocampus plays a critical role in the initial formation of memories (Eichenbaum, 2000), the parahippocampal region is thought to be involved in the intersection between perception and memory, and the translation of material into a more permanent storage in the cortical association areas (Eichenbaum, 2000; Murray and Bussey, 1999). Animal models have shown that the parahippocampal region is important for recognition memory (Brown and Aggleton, 2001; Eichenbaum, 2000), and that selective lesions to the parahippocampal area can severely impair memory (Suzuki et al., 1993). Furthermore, the functions of the medial temporal lobe are highly lateralized and the classic model of material specific memory predicts that lesions in the left hippocampal system impair verbal memory retrieval (Hermann et al., 1997), while those in the right hippocampal system affect non-verbal memory, though these findings are less consistent (Alessio et al., 2004).

In left TLE patients there was a significant correlation between parahippocampal tract FA and material specific memory measures on both the left and right sides. No significant correlations were seen with respect to tract volume in left TLE, and neither volume nor FA in right TLE. The finding of fewer occipital connections in the left TLE group compared with right TLE patients and controls may be significant in this respect (Figs. 2 and 3). Animal models suggest that potential roles for these connections may involve the priming of mesial temporal lobe structures to facilitate the consolidation of visual memory, or enhancing the visual processing of emotionally significant stimuli (Suzuki et al., 1993; Suzuki and Amaral, 1994). Visual cues may be important not only for non-verbal memory, but may also be useful in verbal memory. This could explain the correlation of FA found with verbal and non-verbal memory in left TLE patients. In the right TLE patients the occipital connections did not seem to be as affected, and hence these patients may have been able to use visual cues to aid both verbal and non-verbal memory.

Several other studies have also observed correlations between MRI findings and memory dysfunction in TLE. Some have shown a correlation between left and right hippocampal MRI volumes, and verbal and non-verbal memory respectively (Baxendale et al., 1998; Kalviainen et al., 1997; Lencz et al., 1992). Others have shown correlations between hippocampal T2-signal, or abnormalities of MR spectroscopy and material specific measures (Gadian et al., 1996; Wendel et al., 2001). Lui et al. (Lui et al., 2005) examined 18 TLE patients and found correlations between apparent diffusion coefficients (ADC) in the left and right hippocampi and parahippocampal gyri and, verbal and non-verbal memory respectively. Others have demonstrated correlations between localized diffusion measures and domains of cognitive functioning in diseases as varied as schizophrenia (Nestor et al., 2004), HIV related dementia (Ragin et al., 2005), age related cognitive decline (Charlton et al., 2006), mild cognitive impairment (Rose et al., 2006), and Alzheimer's disease (Yoshiura et al., 2002). There are also studies that have observed that the relationship between the side of pathology and memory dysfunction appears to be more evident in those with left TLE than right TLE. Alessio et al evaluated the relationship between several medial temporal lobe structures and memory in 39 patients, and found a correlation between the degree of left sided hippocampal atrophy and verbal memory deficits, but not between right sided hippocampal atrophy and visual memory deficits (Alessio et al., 2004).

In this study, it also appears that tractography derived parahippocampal FA is a more sensitive marker than volume of the functional integrity of tracts that form the hardwiring of circuits needed for memory (Charlton et al., 2006). Other tractography studies have shown that FA is a more sensitive and robust measure than volume of pathology in white matter tracts (Heiervang et al., 2006). In biological terms the interpretation of reduced anisotropy is complex (Beaulieu, 2002), and depends on the context or disease in which it is found (Alexander et al., 2007). In epilepsy, it may represent neuronal loss, gliosis, and structural disorganisation (Wieshmann et al., 1999).

### The association between hippocampal volume and parahippocampal Volume/FA

No association between hippocampal volume, and tract volume or FA was found in this study. Other studies have reported mixed findings. While Bonhila et al found no correlations between the severity of hippocampal atrophy and grey matter volume in the parahippocampal region (Bonilha et al., 2003), Jutila et al reported positive correlations but only in a subgroup analysis of those patients with the most severe hippocampal atrophy (Jutila et al., 2001). This suggests that despite the fact that the hippocampus and parahippocampal gyrus are significantly interconnected, the structural size relationship between the two structures is not linear. There was also a significant correlation between ipsilateral tract volume and FA in the current study.

### Limitations of Study

Only modest numbers of patients and controls were available for this study, as the scanner used was decommissioned and replaced with one of higher field strength, making comparison with more recent data impossible. These small numbers, particularly in the left TLE group, may have contributed to the pattern of observed changes in left but not right TLE patients. Furthermore, for this reason several morphological and functional factors that can influence cognition and memory in chronic epilepsy were not included as co-variates in the statistical analysis. The pathological basis of TLE was not homogenous throughout the group, and the degree of hippocampal atrophy was varied. Secondly the spatial resolution of the tractography was limited and we did not separately evaluate the entorhinal cortex, parahippocampal cortex, and perirhinal cortex (Duvernoy, 1998). The entorhinal cortex is considered to be the route by which data reaches the hippocampus. The perirhinal, and parahippocampal cortex on the other hand, provide the incoming connections to the entorhinal cortex, conveying information from the polymodal and unimodal cortices (Squire, 1991). A sub-regional analysis is an area that should be explored in future tractography studies.

## Conclusion

This tractography study has shown disruption of the architecture and atrophy of the connections of the parahippocampal gyrus ipsilateral to the seizure focus in patients with refractory TLE, and these structural changes were associated with memory deficits evident on psychometric testing. This information has both diagnostic and prognostic implications. Larger, longitudinal studies at a higher resolution will enable both sub-regional analysis, and the investigation of other factors that may contribute to neuronal loss and structural changes, and subsequent memory impairment in patients with TLE.

## Figures and Tables

**Table 1:** Clinical data for all patients

	Age/gender	Handedness	Age of epilepsy onset (years)	Duration of epilepsy (years)	Seizure types and frequency (per month)	Post-op outcome (ILAE Class)	MRI and pathological diagnosis	Clinical and EEG diagnosis	Right HV (cm^3^)	Left HV (cm^3^)	Hippocampal volume ratio (%)	Total intracranial volume (cm^3^)	AEDs (mg/day)	Language dominance
1	25/F	Right	17	8	CPS 8 SGTC 0.5	1	Left HS	Left TLE	2.73	1.35	49.5	1690	TPR 150 LTG 200	Left
2	37/M	Left	1	36	SPS 12 CPS 4	2	Left HS	Left TLE	3.3	1.88	56	1750	VPA 800 CBZ 800 LVT 2000	Left
3	33/M	Right	1	32	SPS 4 CPS 4	1	Left HS	Left TLE	2.83	2.02	71	1650	PMD 500 CBZ 1200 CLB 10 TPR 175 LVT 4000	Left
4	32/F	Right	22	10	SPS 30 CPS 5 SGTC 0.25	N/A (Died)	Left HS	Left TLE	2.36	1.79	75.9	1530	OXC 1050 TPR 200	Left
5	28/M	Right	3	25	CPS 1	1	Left HS	Left TLE	3.08	1.42	46	1710	LVT 3000 LTG 600	Left
6	31/M	Right	10	21	CPS 50 SGTC 3	1	Left MTL DNET	Left TLE	3.73	3.8	98	1870	CBZ 1200 CLN 1.5 LTG 100	Left
7	34/M	Right	21	13	CPS 5	(No surgery)	Left HS	Left TLE	2.23	1.9	85	1720	LVT 2000 VPA 2000	Left
8	37/F	Right	1	36	SPS12 CPS8 SGTC 1	3	Left HS, L fusiform gyrus ganglioglioma	Left TLE	2.8	2.38	85	1350	CBZ 1000 CLB 10	Left
9	25/M	Right	5	20	CPS 3	N/A (No surgery)	Right HS	Right TLE	1.84	2.35	78	1500	PHT 200 LVT 3000	Left
10	47/M	Right	13	34	CPS 1	2	Right HS	Right TLE	1.04	2.77	37.5	1460	LVT 500 PHT 300 CBZ 800	Left
11	41/F	Right	14	27	CPS 4	1	Right superior temporal FCD	Right TLE	2.6	2.67	97	1520	TGB 15	Left
12	44/F	Right	14	30	CPS 4	1	Right HS	Right TLE	2.61	3.03	86	1550	LVT 750 PHT 400 CLB 10	Left
13	44/M	Right	14	30	CPS 4	?1	Right HS	Right TLE	2.78	3.05	91	1760	CBZ 800 LVT 1000	Left
14	36/M	Left	15	21	SPS 6 CPS 6 SGTC 3	1	Right MTL glioma	Right TLE	3.12	2.77	89	1520	CBZ 1600 CLB 20 LTG 400	Right
15	46/F	Right	8	38	CPS 5	1	Right HS	Right TLE	1.75	2.69	65	1470	TPR 600 PMD 1000 OXC 2400	Left
16	31/F	Right	19	12	CPS 2	4	Right HS	Right TLE	2.3	2.56	89.75	1400	CBZ 1600 LVT 1000	Left
17	29/M	Right	9	20	CPS 30 SGTC 4	3	Right HS	Right TLE	2.27	2.98	76	1630	VPA 2400	Left
18	22/M	Left	18	4	CPS 3 SGTC 0.33	1	Right MTL DNET	Right TLE	2.49	2.55	97.6	1680	VPA 2000 GBP 600	Left

M = male, F = female, SPS = simple partial seizure, CPS = complex partial seizure, SGTC = secondarily generalised seizure, N/A = not available, HS = hippocampal sclerosis, MTL = mesial temporal lobe, DNET = dysembryoblastic neuroepithelial tumour, TLE = temporal lobe epilepsy, TPR = topiramate, LTG = lamotrigine, VPA = sodium valproate, CBZ = carbamazepine, LVT = levetiracetam, PMD = primidone, CLB = clobazam, OXC = oxcarbamazepine, CLN = clonazepam, PHT = phenytoin, TGB = tiagabine, GBP = gabapentin.

**Table 2:** Neuropsychological test results of patients

Group	Mean VIQ	Mean PIQ	Mean VL	Mean DL
Left TLE	82	90	59	59
Right TLE	89	97	65	63

VIQ = verbal IQ, PIQ = performance IQ, VL = verbal learning, DL = design learning.

**Table 3:** Asymmetry indices of FA and volume, and mean absolute values of FA and volume in controls and patients

	Controls	Left TLE	Right TLE
Left volume ± SE (mm^3^)	7130 ± 605	5756 ± 810	7030 ± 653
Right volume ± SE (mm^3^)	6791 ± 959	7424 ± 1052	6411 ± 1019
AI Volume ± SE (%)	− 8.58 ± 12.50	26.22 ± 7.06^⁎^	− 17.72 ± 14.2
Left FA ± SE	0.29 ± 0.05	0.26 ± 0.01^⁎^	0.27 ± 0.01
Right FA ± SE	0.29 ± 0.01	0.29 ± 0.01	0.26 ± 0.01
AI FA ± SE (%)	− 0.99 ± 3.26	11.80 ± 4.67^⁎^	− 5.26 ± 3.70

AI = asymmetry index = [100 × (right − left)] / [(right + left)/2]. ^⁎^ = *p* ≤ 0.05 compared with control subjects.
